# Effect of Dopamine Therapy on Nonverbal Affect Burst Recognition in Parkinson's Disease

**DOI:** 10.1371/journal.pone.0090092

**Published:** 2014-03-20

**Authors:** Julie Péron, Didier Grandjean, Sophie Drapier, Marc Vérin

**Affiliations:** 1 Swiss Center for Affective Sciences, University of Geneva, Geneva, Switzerland; 2 ‘Neuroscience of Emotion and Affective Dynamics’ laboratory, Department of Psychology, University of Geneva, Geneva, Switzerland; 3 ‘Behavior and Basal Ganglia’ research unit (Host Team 4712), University of Rennes 1 and Pontchaillou Hospital, Rennes University Hospital, Rennes, France; 4 Neurology Unit, Pontchaillou Hospital, Rennes University Hospital, Rennes, France; University of Cincinnati, United States of America

## Abstract

**Background:**

Parkinson's disease (PD) provides a model for investigating the involvement of the basal ganglia and mesolimbic dopaminergic system in the recognition of emotions from voices (i.e., emotional prosody). Although previous studies of emotional prosody recognition in PD have reported evidence of impairment, none of them compared PD patients at different stages of the disease, or ON and OFF dopamine replacement therapy, making it difficult to determine whether their impairment was due to general cognitive deterioration or to a more specific dopaminergic deficit.

**Methods:**

We explored the involvement of the dopaminergic pathways in the recognition of nonverbal affect bursts (onomatopoeias) in 15 newly diagnosed PD patients in the early stages of the disease, 15 PD patients in the advanced stages of the disease and 15 healthy controls. The early PD group was studied in two conditions: ON and OFF dopaminergic therapy.

**Results:**

Results showed that the early PD patients performed more poorly in the ON condition than in the OFF one, for overall emotion recognition, as well as for the recognition of anger, disgust and fear. Additionally, for anger, the early PD ON patients performed more poorly than controls. For overall emotion recognition, both advanced PD patients and early PD ON patients performed more poorly than controls. Analysis of continuous ratings on target and nontarget visual analog scales confirmed these patterns of results, showing a systematic emotional bias in both the advanced PD and early PD ON (but not OFF) patients compared with controls.

**Conclusions:**

These results i) confirm the involvement of the dopaminergic pathways and basal ganglia in emotional prosody recognition, and ii) suggest a possibly deleterious effect of dopatherapy on affective abilities in the early stages of PD.

## Introduction

Emotional prosody is defined as modifications in segmental and suprasegmental speech parameters during an emotional episode (for a review, see [1]). fMRI and patient lesion studies have allowed researchers to delineate a distributed neural network involved in the recognition of emotional prosody, encompassing the primary and secondary auditory areas, the superior temporal sulcus and gyrus, and the amygdala (e.g., [2]). Modulations in activity within anterior regions such as the orbitofrontal cortex and inferior frontal areas have been described in response to emotional prosody (e.g., [3]). Beyond these regions, researchers have also reported the involvement of the basal ganglia (BG), particularly the caudate nucleus and putamen [4], but also the subthalamic nucleus (STN) [5]–[7], in the processing of emotional prosody.

Parkinson's disease (PD), a neurodegenerative disorder affecting the nigrostriatal and mesocorticolimbic dopaminergic systems, offers an opportunity to study the influence of the BG and dopaminergic pathways on emotional prosody processing (for a review, see [8], [9]). To date, studies have found that adults with PD display impaired recognition of (negatively valenced) emotional prosody when compared with matched healthy controls (HC), reinforcing the hypothesis of BG involvement in the processing of emotional prosody (for a review, see [8], [9]). However, the profiles of the patients included in these studies were quite heterogeneous (sociodemographically, as well as clinically and cognitively) [9], making it difficult to determine whether these deficits are due to general cognitive deterioration or to a more specific dopaminergic deficit related to PD. In particular, none of these studies deliberately included PD patients at different stages of the disease or compared patients ON and OFF dopamine replacement therapy (DRT). As recently explained by MacDonald and colleagues [10], PD could represent a powerful study model, providing these variables (i.e., disease duration and DRT) are controlled for, as it would enable researchers to explore the involvement in nonmotor functions of i) the substantia nigra and dorsal striatum, and ii) ventral tegmental area (VTA)-innervated regions such as the ventral striatum and the prefrontal and limbic cortices. The early stages of PD are characterized by the degeneration of the substantia nigra, leading to a restricted supply of dopamine in the dorsal striatum, but VTA-innervated regions are thought to be relatively spared. As the pathology progresses, VTA degeneration and the dopamine deficiency of its efferent structures increase. Although DRT improves motor symptoms at every stage of the disease, the effects of this treatment on nonmotor functions, such as cognitive processes, differ according to the stage of the disease. Like motor functions, the cognitive functions that rely on the dorsal striatum seem unimpaired in early PD and are remediated by DRT [11]–[13]. By contrast, while the cognitive functions that depend upon VTA-innervated regions such as the ventral striatum are also thought to be unimpaired in the early stages of PD, they are worsened by DRT [13], [14], owing to dopamine toxicity, in an effect known as the *dopamine overdose effect* [15]. Recently, using a reward learning paradigm, MacDonald and colleagues [10] confirmed that i) patients with both advanced and early PD display poorer cognitive performances than HC, and ii) DRT only worsens cognitive performances in the early stages of the disease. To date, and to the best of our knowledge, the dopamine overdose effect has never been tested in emotion processing. The only study to have compared PD patients' recognition of emotional prosody in ON versus OFF DRT conditions is that of Breitenstein and colleagues [16]. In their study, they included a group of de novo (i.e., OFF DRT) PD patients, a group of advanced PD patients (ON DRT), and an HC group. Results showed that the advanced PD patients performed significantly worse than the HC group, but there was no significant difference either between the early PD patients and HC or between the early PD and advanced PD patients. In this study, however, the ON versus OFF dopa conditions were compared in an intergroup design, with patients in early (OFF) and late (ON) stages of the disease. In addition, there was no a posteriori verification that the early PD patients responded well to DRT, making it impossible to confirm their PD diagnosis [17].

Accordingly, we do not yet know whether the emotional prosody recognition deficits observed in PD arise from the cortical diffusion of the lesions as the disease progresses, or from specific dopaminergic depletion. The aim of the present study was thus to increase current understanding of BG and dopaminergic pathway involvement in the recognition of emotional prosody. We therefore used an original paradigm featuring the recognition of emotional bursts (i.e., onomatopoeias) to compare newly diagnosed PD patients ON or OFF DRT, and patients with advanced pathology (all receiving DRT) with HC. Based on the recent findings of MacDonald and colleagues [10] in the cognitive domain, together with results pointing to emotional prosody disturbances in PD, we predicted that the PD patients ON DRT would perform more poorly than the HC in the emotional prosody task, especially for negatively valenced emotions, whichever stage of the disease (early or advanced) they were at (Hypothesis 1), and the early PD patients would display poorer recognition of the negative emotional bursts in the ON condition than in the OFF one (Hypothesis 2).

## Experimental Procedures

### 1. Participants

Two groups of patients with PD at different stages of the disease (early and advanced PD) and an HC group took part in the study (*n* = 15 in each group). The characteristics of the three groups are set out in Table 1.

**Table 1:** Clinical, demographic, and neuropsychological data (mean ± *SD*) for the two PD groups and the HC group.

	Early PD (*n* = 15)				Advanced PD (*n* = 15)		HC (*n* = 15)		Statistical value	*p* value
	ON DRT		OFF DRT							
	Mean	*SD*	Mean	*SD*	Mean	*SD*	Mean	*SD*		
**Sex (F/M)**	10F/5M	-			10F/5M	-	10F/5M	-	-	-
**Age (years)**	60.3	7.3			59.5	8.6	55.9	7.8	1.26§	.3
**Education (years)**	12.6	4.4			13.8	3.6	13.8	2.3	0.63§	.5
**Handedness (R/L)**	15 R	-			15 R	-	15 R		-	-
**Disease duration (years post-onset)**	2.8	1.2			11.1	3.4	-	-	−8.92#	<.001*
**DRT (mg)**	437.3	229.1			974.7	477.5	-	-	−3.91#	<.01*
**H&Y rating score ON**	0.6	0.7			1.3	0.8	-	-	−2.49#	<.01*
**H&Y rating score OFF**	1.3	0.6			2.4	1.0	-	-	−3.44##	<.01*
**S&E rating score (%) ON**	93.6	6.3			91.5	8.0	-	-	0.73#	.5
**S&E rating score (%) OFF**	88.6	7.7			70.0	22.0	-	-	2.97##	<.01*
**MADRS**	6.1	5.0			7.6	8.0	1.8	2.5	3.38§	<.05*
**STAI A**	39.0	12.0			43.0	14.3	39.6	12.2	1.06§	.4
**STAI B**	38.0	6.1			39.6	13.4	35.7	10.8	5.56§	<.05*
**MMSE**	-	-	-	-	-	-	29.1	0.83	-	-
**Mattis (MDRS)**	139.5	4.8	139.6	4.8	140.9	2.6	141.6	2.2	1.41§	.3 - .3
**Stroop interference test**	−1.9	11.8	-	-	6.2	7.0	5.1	10.8	2.95§	.06
**TMT A (in s)**	42.5	12.7	-	-	43.3	15.1	43.6	16.5	0.21§	.9
**TMT B (in s)**	104.3	36.8	-	-	104.3	36.8	99.2	46.3	0.25§	.8
**TMT B-A (in s)**	59.0	29.2	-	-	50.4	30.7	55.6	38.0	0.26§	.7
**Categorical verbal fluency (2 min)**	26.3	8.2	-	-	33.6	12.3	33.3	8.8	2.60§	.08
**Phonemic verbal fluency (2 min)**	19.9	7.4	-	-	23.3	6.9	21.2	6.4	0.9§	.4
**Action (verb) fluency (1 min)**	14.7	5.2	-	-	16.3	7.6	14.7	5.2	1.91§	.2
**MCST (no. categories)**	5.6	1.1	-	-	5.8	0.4	5.9	0.3	0.78§	.5
**MCST (no. errors)**	4.7	7.9	-	-	3.8	3.8	2.3	2.2	0.71§	.5
**MCST (no. perseverative errors)**	2.0	4.8	-	-	0.8	1.3	0.3	0.6	1.23§	.3

Differential effects between the three groups are reported (single-factor ANOVA and *t* tests for two independent groups).

*Abbreviations:* DRT: dopamine replacement therapy; HC: healthy controls; H&Y: Hoehn and Yahr; HC: healthy controls; MADRS: Montgomery-Asberg Depression Rating Scale; MCST: Modified Wisconsin Card Sorting Test; MMSE: Mini-Mental State Examination; PD: Parkinson's disease; S&E: Schwab and England; *SD*: standard deviation; STAI: State-Trait Anxiety Inventory; TMT: Trail Making Test.

§ Comparisons (single-factor ANOVA) were performed between the early PD ON, advanced PD and HC groups.

# Comparisons (*t* test for two independent groups) were performed between the early PD ON and advanced PD groups.

## Comparisons (*t* test for two independent groups) were performed between the early PD OFF and advanced PD groups.

* Statistically significant (*p*<0.05).

All the patients met the clinical criteria of the United Kingdom PD Society brain bank for idiopathic PD [17].

Disease severity was rated using the revised Hoehn and Yahr (H&Y) disability scale [18] and the Schwab and England (S&E) daily living activities scale [19]. The early PD group was examined into two conditions: ON and OFF daily DRT (levodopa preparations and/or dopamine receptor agonists). Intake was defined as the *levodopa equivalent dose*, calculated on the basis of correspondences adapted from Tomlinson and colleagues [20]. All the early PD patients were stable on their medication and good responders. For the OFF DRT condition, they were asked to abstain from taking their medication the night before the assessment (i.e., their last medication was taken at about 3 pm and the assessment was performed 18–20 hours later). All the advanced and early ON patients were on levodopa and non-controlled release dopamine receptor agonists (ropinirole and/or pramipexole). The plasma half-life of levodopa is 1–2 hours [21], that of ropinirole is 6 hours, and that of pramipexole 8 hours [22]. All the advanced PD patients remained on DRT throughout the procedure.

The HC group consisted of healthy individuals who had no history of neurological disease, head injury or alcohol abuse, and no signs of dementia, as attested by their scores on the Mini-Mental State Examination (MMSE) [23].

All three groups were of comparable age and education level (Table 1).

After the participants had been given a complete description of the study, they all provided their written informed consent, and the study was conducted in accordance with the Declaration of Helsinki. The study protocol was approved by the ethics committee of the hospital where all the data were acquired (Neurology Unit of Pontchaillou Hospital (Rennes University Hospital), France, Prof. M. Vérin).

### 2. Neuropsychological and psychiatric screening

As described elsewhere [6], [24]–[27], a short neuropsychological and psychiatric battery was administered to the participants prior to the vocal emotion recognition sessions (see Table 1). This battery included the Mattis Dementia Rating Scale (MDRS) [28] and a series of tests assessing frontal executive functions, including Nelson's modified version of the Wisconsin Card Sorting Test (MCST) [29], the Trail Making Test (TMT) [30], the Categorical and Literal Fluency test [31], the Action (Verb) Fluency task [32], and the Stroop test [33]. Depression was assessed using the Montgomery-Asberg Depression Rating Scale [34]. The MADRS was chosen because of the predominance of cognitive items over somatic ones, thus limiting interference with Parkinson's symptoms. Finally, the State-Trait Anxiety Inventory (STAI) [35] was used to assess anxiety. The early PD patients only underwent the MDRS in the OFF condition.

### 3.Vocal emotion recognition procedure

#### 3.1 Vocal emotion recognition stimuli

A set of vocal stimuli consisting of nonverbal affect bursts (onomatopoeias) was played to all participants. These stimuli were taken from the Montreal Affective Voices (MAV) database developed and validated by Belin and colleagues [36].

The onomatopoeias were produced by 10 different actors (5 women and 5 men) in seven different prosodies (anger, fear, happiness, neutral, disgust, surprise, and sadness), making a total of 70 vocal stimuli. The mean (±SD) duration of the stimuli was 1084 ms (±722 ms) and the mean (± SD) energy of the stimuli was 73.4 dB (±9.6 dB).

#### 3.2 Vocal emotion recognition procedure

All the stimuli were played binaurally via stereo headphones using an Authorware program designed especially for this study [6], [27]. Participants sat comfortably in a quiet room, in front of the computer, and looked at a fixation cross while listening to the stimuli. They were told that they would hear meaningless speech uttered by male/female actors and that these actors would express emotions via their utterances. Participants were required to listen to each stimulus, after which they were asked to rate its emotional content on a set of visual analog scales displayed simultaneously on the computer screen. More specifically, participants were instructed to judge the extent to which the different emotions were expressed on visual analog scales ranging from “*Not at all*” to “*Very much*”. There were seven scales: one for each prosody (anger, fear, happiness, neutral, disgust, surprise and sadness). Participants were told they could listen again to each stimulus as many as six times, by clicking on a button on the computer interface. They were played two examples in order to familiarize themselves with the task. An example of the computer interface used for the recognition of emotional prosody (onomatopoeias) task is provided in Appendix S1.

As described elsewhere [6], [24], [26], [37], and in order to avoid a list effect between the ON and OFF DRT conditions in the early PD patient group, the stimuli were counterbalanced. In the ON condition, half the early PD patients were assessed with Version A of the vocal emotion recognition task and half with Version B. In the OFF condition, the former were assessed with Version B and the latter with Version A. The same counterbalancing method was applied to the advanced PD group and the HC group, which were both divided into two subgroups, with one subgroup being assessed with Version A and the other with Version B. The entire protocol was completed in a single 90-min session. The early PD patients underwent a second session in the OFF (or ON) condition. The ON versus OFF DRT sessions were randomized.

#### 3.3 Audiometric screening procedure

To ensure that the participants had normal hearing, we administered a standard audiometric screening procedure (AT-II-B audiometric test) to measure tonal and vocal sensitivity. None of the patients included in the study wore hearing aids or had a history of tinnitus or a hearing impairment.

### 4. Statistical analysis

The sociodemographic, neuropsychological and psychiatric variables of the three groups were first compared using a single-factor analysis of variance (ANOVA). Whenever the ANOVA yielded a significant difference, pairwise *t* tests for two independent groups were carried out to determine which groups differed from one another. Within-group comparisons were conducted in the early PD group, to compare the OFF versus ON DRT scores on the MDRS, using *t* tests for dependent groups.

For the vocal emotion recognition data, we constructed two generalized linear models (GLMs) to address our two operational hypotheses: 1) a model including the early PD ON, advanced PD and HC patients (Hypothesis 1); and 2) a model including the early PD patients in the ON versus OFF conditions (Hypothesis 2). The participants' responses were then investigated using two complementary methods adapted to the experimental paradigm. First, we compared their performances on *categorical ratings*, in terms of percentages of correct responses. A response was deemed to be correct when a participant provided a higher rating on the target scale (e.g., the *Anger* scale when the stimulus was *anger*) than on all the other (nontarget) scales. Second, we compared their performances on *continuous ratings* for each type of prosody, on the basis of a) target scales and b) nontarget scales. Table 2 describes the detailed statistical methodology.

**Table 2:** Synopsis of statistical analyses.

**1. Sociodemographic, neuropsychological and psychiatric data**	- Single-factor analysis of variance (ANOVA) for the three groups, i.e. early PD ON, advanced PD, and HC; if significant, pairwise *t* tests for two independent groups.	
	- Pairwise *t* tests for two dependent groups in the early PD group to compare OFF vs. ON dopa scores on the Mattis Dementia Rating Scale	
**2. Vocal emotion recognition data**		
***2.1 Categorical ratings***	*Model 1* - Intergroup analyses including the early PD ON, advanced PD and HC groups	Single-factor ANOVA. Whenever the ANOVA yielded a significant difference, pairwise *t* tests were conducted for two independent groups
	*Model 2* - Intragroup analyses including the early PD patients in the OFF vs. ON DRT conditions	Pairwise *t* tests for two dependent groups
***2.2 Continuous ratings***	*Model 1* - Intergroup analyses including the early PD ON, advanced PD and HC groups	Repeated-measures ANOVA with two within-participants factors-prosody (7 levels) and scale (7 levels)-and one between-participants factor-group (early PD ON, advanced PD and HC; 3 levels). If the results of the latter were significant, to investigate the effects in greater detail, contrasts were performed between the three groups (early PD ON vs. advanced PD, early PD ON vs. HC, and advanced PD vs. HC) for each type of prosody and each rating scale.
	*Model 2* - Intragroup analyses including the early PD patients in the OFF vs. ON DRT conditions	Repeated-measures ANOVA with three within-participants factors - condition (2 levels), prosody (7 levels) and scale (7 levels). If the results of the latter were significant, to investigate the effects in greater detail, contrasts were performed between the two conditions (early PD ON vs. early PD OFF) for each type of prosody and each rating scale.
***2.3 Version A vs. Version B***	Chi-square (χ^2^)	
**3. Correlations between neuropsychological, sociodemographic and clinical variables and vocal emotion recognition variables**	Pearson correlation coefficient	

Versions A and B of the emotional prosody recognition task were compared using the χ^2^ test in the HC group.

We then computed Pearson's correlation coefficients between the neuropsychological, sociodemographic and clinical variables and the emotional prosody recognition variables.

The level of statistical significance was set at *p* = 0.05, except for the GLM post hoc comparisons of emotional prosody performances for which we did not have any a priori hypotheses (i.e., positively valenced emotions and neutral utterances), and the Pearson correlations, where the *p* value was adjusted for multiple comparisons.

Statistical analyses were performed using Statistica 12.

## Results

### 1. Clinical assessment (Table 1)

Significant differences were found between the early PD and advanced PD patients on the H&Y (early PD ON and OFF dopa) and S&E (early PD OFF dopa only).

### 2. Neuropsychological and psychiatric assessments (Table 1)

Results revealed a significant difference between the three groups on the MADRS and STAI-B. Pairwise comparisons revealed that the early PD patients scored significantly higher than the HC (MADRS: *t* = 2.68, *p* = .01; STAI-B: *t* = 3.69, *p* = .001), as did the advanced PD group (MADRS: *t* = 2.36, *p* = .03; STAI-B: *t* = 2.63, *p* = .01), but there was no significant difference between the early PD and advanced PD patients (MADRS: *t* = −0.54, *p* = .6; STAI-B: *t* = −0.25, *p* = .8).

Results showed that there was no significant difference between the three groups on any of the neuropsychological variables.

### 3. Recognition of vocal emotions

#### 3.1 Model 1 – Comparisons between the early PD ON, advanced PD and HC groups (Tables 3 & 4)

The *first statistical model* we built included the early PD ON, advanced PD and HC groups, in order to test our prediction that, whichever stage of the disease they were at (early or advanced), the PD patients on DRT would perform more poorly than the HC on the emotional prosody task.

The first level of analysis, consisting of an investigation of categorical ratings (Table 3), seemed to reveal two different patterns of results.

**Table 3:** Percentage of correct responses (*SD*) for categorical ratings in the emotional prosody recognition task for early PD patients in the ON and OFF DRT conditions, advanced PD patients, and HC.

	Early PD (*n* = 15)				Advanced PD (*n* = 15)		HC (*n* = 15)		*df*	Stat. val. (*F*)§	*p* value§
	ON DRT		OFF DRT								
	Mean	±*SD*	Mean	±*SD*	Mean	±*SD*	Mean	±*SD*	-	-	-
**Anger**	22.67	22.51	42.67	30.11	37.33	21.20	46.61	20.90	2	4.72	.01*
**Disgust**	49.33	12.80	61.33	23.26	48.00	12.65	62.67	24.92	2	3.13	.05
**Fear**	45.67	30.57	62.67	26.04	41.33	23.26	64.00	27.46	2	2.87	.07
**Happiness**	87.00	19.68	92.23	11.31	86.67	27.95	96.33	6.04	2	1.25	.3
**Neutral**	82.57	15.67	84.33	19.22	83.00	17.23	86.67	25.82	2	<1	.8
**Sadness**	77.57	21.22	85.33	19.22	80.00	18.51	86.67	20.93	2	<1	.5
**Surprise**	77.33	14.86	84.00	18.82	74.67	20.66	81.33	19.22	2	<1	.6
**Total**	63.43	11.34	73.52	14.06	64.57	9.34	75.05	7.60	2	6.75	.003*

*Abbreviations:* HC: healthy controls; PD: Parkinson's disease; *SD*: standard deviation; DRT: dopamine replacement therapy.

The statistical values (Stat. val.), degrees of freedom (*df*) and *p* values between the three independent groups are reported (single-factor ANOVA).

§ Comparisons (ANOVA) were performed between the early PD ON, advanced PD and HC groups.

* Significant (*p*<0.05).

- First, for the overall recognition score, both the advanced PD and early PD ON patients performed more poorly than the HC (advanced PD vs. HC: *t* = −3.37, *p*<.01; early PD ON vs. HC: *t* = −3.30, *p*<.01), whereas there was no significant difference between the advanced PD and early PD ON patients (*t* = 0.30, *p* = .8).
- Second, for the *anger* recognition subscore, impairment seemed to be restricted to the early PD ON group, with pairwise comparisons revealing that the early PD ON patients performed more poorly than the HC (*t* = −3.02, *p*<.01). There was a trend toward significance in the difference between the advanced PD and early PD ON patients (*t* = 1.83, *p* = .07), but no difference between the advanced PD patients and the HC (*t* = 1.21, *p* = .2).

The other comparisons were not significant (see Table 3).

The second level of analysis, consisting of an investigation of continuous ratings (Table 4), allowed us to probe our data in greater depth. Overall, analysis revealed a Group×Emotion×Scale interaction, *F*(72, 1512) = 1.46, *p*<.01, showing that the early PD ON patients, advanced PD patients, and HC displayed different patterns of responses to the different scales and different emotions. In order to investigate these effects in greater detail, we ran Group×Scale interaction analyses for each separate prosody. These analyses revealed that there were no Group×Scale interaction effects for the *happiness*, *sadness*, *surprise*, *disgust* and *neutral* prosodies (*F*<1 for all comparisons). There were, however, significant interactions for *anger*, *F*(12, 252) = 3.10, *p*<.001, and *fear*, *F*(12, 252) = 2.38, *p*<.01.

**Table 4:** Mean ratings (standard deviations) on the visual analog scales in the emotional prosody task (onomatopoeias) for early PD patient in the ON and OFF DRT conditions, advanced PD patients, and HC.

Early PD group in the ON DRT condition (*n* = 15)							
Emotion	*Happi.* scale	*Fear* scale	*Sad.* scale	*Anger* scale	*Neutral* scale	*Disgust* scale	*Surp.* scale
***Anger***	5.78±8.23	27.12±17.99** ##	1.22±2.45	19.19±16.11	4.34±10.62	20.29±21.98	13.32±19.61
***Disgust***	6.53±8.01	18.16±17.05	1.02±2.48	3.12±6.35	5.54±9	36.69±20.8	6.97±9.66
***Fear***	0.11±0.17	33.16±19.59	0.57±1.53	10.96±15.37** †	1.89±3.81	30.96±17.64* ##	8.72±16.41
***Happiness***	60.34±17.72	0.54±1.5	0.15±0.13	0.12±0.17	3.01±5.72	11.77±11.76	0.54±1.9
***Neutral***	0.16±0.2	0.55±1.76	1.68±2.95	0.93±3.14	50.16±33.63	3.02±7.61	4.09±1.42
***Sadness***	5.86±6.55	7.02±8.22	55.12±19.99	0.39±0.92	3.34±6.1	3.14±6.87	1.25±3.68
***Surprise***	0.99±2.09	2.35±4.21	2.99±6.09	0.6±1.68	1.15±2.33	13.07±16.21	53.71±18.47

Early PD group in the OFF DRT condition (*n* = 15)							
Emotion	*Happi.* scale	*Fear* scale	*Sad.* scale	*Anger* scale	*Neutral* scale	*Disgust* scale	Surp. scale
***Anger***	5.55±7.12	18.13±14.67	0.32±0.32	25.79±22.83	1.11±3.57	23.63±19.64	15.88±19.37
***Disgust***	2.81±5.83	14.35±15.46	0.57±1.37	5.83±7.15	5.01±6.84	40.65±21.15	8.98±13.9
***Fear***	2.34±6.1	37.45±19.61	2.46±3.97	2.58±5.38	6.35±8.48	24.91±20.97	7.35±10.42
***Happiness***	61.39±17.19	0.41±1.11	0.19±0.2	1.31±3.32	3.28±7.21	11.99±15.97	0.36±0.89
***Neutral***	0.14±0.22	1.37±3.35	2.56±3.17	0.51±0.9	53.18±14.04	5.53±3.95	3.6±6.19
***Sadness***	4.66±5.32	7.02±12.66	55.8±17.29	3.76±8.3	2.15±2.86	2.45±4.82	4.74±7.69
***Surprise***	1.4±3.56	4.48±8.65	2.34±4.84	1.51±3.67	5.61±8.01	17.17±22.05	52.31±20.49

Advanced PD group (*n* = 15)							
Emotion	*Happi.* scale	*Fear* scale	*Sad.* scale	*Anger* scale	*Neutral* scale	*Disgust* scale	*Surp.* scale
***Anger***	3.82±7.14	11.26±7.63	1.18±2.57	23.13±11.51	2.89±6.97	23.32±11.84*	8.94±10.5
***Disgust***	4.57±7.69	16.29±11.76	1.3±3.16	1.78±3.46	4.96±6.56	32.64±15.36	7.61±7.76
***Fear***	1.13±2.48	33.57±20.12	1.31±3.34	32.55±21.03*	4.27±5.54	14.13±11.75	7.44±8.33
***Happiness***	64.84±19.97	0.42±0.24	2.26±5.68	0.62±0.11	3.03±8.09	4.78±8.74	0.74±2.36
***Neutral***	4.6±5.97	0.54±1.97	0.65±1.01	0.22±0.42	51.05±16.95	6.43±4.51	1.61±3.9
***Sadness***	9.93±8.06	3.93±429	56.55±21.16	0.44±0.86	1.51±7.2	1.14±3.38	0.34±0.68
***Surprise***	0.21±0.19	1.07±1.96	2.95±3.79	1.86±3.77	1±1.98	12.65±15	49.15±18.26

HC group (*n* = 15)							
Emotion	*Happi.* scale	*Fear* scale	*Sad.* scale	*Anger* scale	*Neutral* scale	*Disgust* scale	*Surp.* scale
***Anger***	5.07±4.84	9.96±7.36	1.36±2.52	26.21±11.76	2.20±4.43	9.11±16.89	12.03±14.05
***Disgust***	4.73±5.14	14.54±7.74	0.73±1.77	1.46±3.17	2.73±3.38	32.31±16.39	6.26±6.44
***Fear***	1.57±3.42	35.15±11.5	0.57±1.65	1.53±4.39	2.68±3.94	18.15±14.98	2.63±7.5
***Happiness***	62.91±8.31	0.21±0.17	1.62±5.64	0.28±0.31	0.18±0.17	0.88±2.35	0.24±0.21
***Neutral***	0.66±1.35	0.70±1.65	0.28±0.34	0.22±0.21	42.11±25.63	3.21±5.77	0.22±0.26
***Sadness***	6.51±7.67	1.24±3.13	56.38±17.68	0.35±0.64	0.22±0.19	0.26±0.16	0.27±0.3
***Surprise***	2.09±3.54	1.1±2.47	2.35±4.78	1.68±3.62	0.62±1.71	6.8±6.75	42.46±15.07

*Abbreviations:* Happi.: Happiness; HC: healthy controls; PD: Parkinson's disease; DRT: dopamine replacement therapy; Sad.: Sadness; *SD*: standard deviation; Surpr.: Surprise.

* Significant if *p*<0.05 and

** significant if *p*<0.01 compared with healthy controls (HC).

# Significant if *p*<0.05 and

## significant if *p*<0.01 compared with advanced PD group.

† Significant if *p*<0.05 and

†† significant if *p*<0.01 compared with early PD group in the OFF DRT condition.

**Figure 1:**
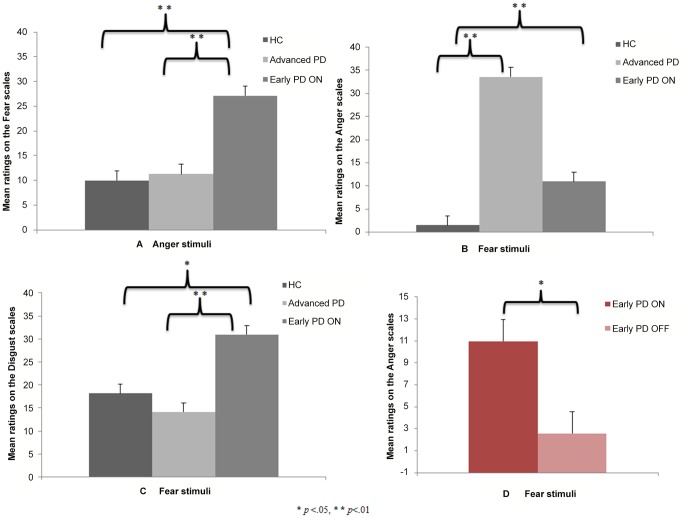
Mean ratings (and standard errors) (A) across all three groups (early PD ON, advanced PD and HC) on the *Fear* scale when the stimulus was *anger*, (B) across all three groups (early PD ON, advanced PD and HC) on the *Anger* scale when the stimulus was *fear*, (C) across all three groups (early PD ON, advanced PD and HC) on the *Disgust* scale when the stimulus was *fear*, and (D) provided by early PD patients in the ON versus OFF conditions on the *Anger* scale when the stimulus was *fear*.

For *fear* and *anger*, contrasts were performed between the three groups for each type of prosody and a) the value on the target scale (e.g., the *Anger* scale) corresponding to the participants' ratings of the relevant stimulus (e.g., *anger*), and b) the values on the nontarget scales, that is, those that did not correspond to the stimulus emotion (e.g., the *Fear* scale for the *anger* stimulus). Contrasts were also run on the nontarget scales in order to investigate the patterns of confusion between the different emotions. These results are extensively described in Table 4 and illustrated in Figure 1 (Panels A, B & C). These analyses revealed the same two patterns of results as for the categorical analyses.

- Both the advanced PD and early PD ON patients gave significantly higher ratings on the *Anger* scale when they listened to *fear* stimuli (Fig. 1 Panel B, & Table 4). Although the contrast between the early PD ON patients and the HC failed to reach significance, the ratings they gave on the *Disgust* scale when they listened to *anger* stimuli seemed to reflect a similar pattern of results (Table 4).
- Emotional bias seemed only to concern the early PD ON patients, who provided significantly higher ratings on the *Fear* scale when they listened to *anger*, and significantly higher ratings on the *Disgust* scale when they listened to *fear* than both the advanced PD patients and HC (Fig. 1 Panels A & C, & Table 4). In these contrasts, there was no significant difference between the advanced PD patients and the HC.

#### 3.2 Model 2 – Comparisons between the early PD patients in the OFF vs. ON conditions (Tables 3 & 4, Fig. 1)

The *second model* included the early PD patients in the ON versus OFF conditions, as we postulated that the toxicity of dopatherapy for emotional functions in the early stages of the disease would mean that the early PD patients performed more poorly on the recognition of emotional bursts in the ON condition than in the OFF one.

The first level of analysis, consisting of an investigation of categorical ratings (Table 3), showed a significant difference between the early PD patients in the ON versus OFF conditions for the overall recognition score (*t* = 3.13, *p*<.01) and the *anger* (*t* = 2.29, *p* = .04), *disgust* (*t* = 2.55, *p* = .02), and *fear* (*t* = 2.48, *p* = .03) recognition subscores, revealing that the early PD patients performed more poorly in the ON condition than in the OFF one. No significant difference was found for the other prosodies (*happiness*: *t* = 1.17, *p* = .3; *sadness*: *t* = 1.00, *p* = .3; *surprise*: *t* = 1.43, *p* = .2; *neutral*: *t* = 0.38, *p* = .7).

The second level of analysis, consisting of an investigation of continuous ratings (Table 4 & Fig. 1), revealed a Group×Prosody×Scale interaction, *F*(36, 504) = 1.57, *p* = .02, showing that the early PD patients displayed different patterns of responses to the different scales and different prosodies in the ON versus OFF conditions. In order to investigate these effects in greater detail, we ran Group×Scale interaction analyses for each separate prosody. These revealed that there were no Group×Scale interaction effects for the *happiness*, *sadness*, *surprise*, *disgust*, *anger*, or *neutral* prosodies (*F*<1 for all comparisons). For *fear*, however, analyses revealed a trend toward significance for the Group×Scale interaction, *F*(6, 84) = 2.20, *p* = .05. Given this trend, and because we had formulated specific hypotheses concerning the performances of the early PD patients in the ON versus OFF conditions (Hypothesis 2), we ran contrasts on each scale, revealing the following patterns of performances. When the stimulus was *fear* and the scale *Anger*, there was a significant difference between the ON and OFF conditions, *F*(1, 14) = 4.73, *p* = .04 (Fig. 1 Panel D). There were no differences when the scale was *Fear* (target scale), *F*<1, *Happiness*, *F*(1, 14) = 2.68, *p* = .1, *Sadness*, *F*(1, 14) = 3.51, *p* = .08, *Disgust*, *F*(1, 14) = 1.77, *p* = .2, *Surprise*, *F*<1, or *Neutral*, *F*(1, 14) = 3.76, *p* = .07. These results are extensively described in Table 4 and illustrated in Figure 1 Panel D.

#### 3.3 Additional analyses–Comparisons between the early PD patients in the OFF condition and HC

As we observed, in line with our hypothesis, that the early PD patients performed worse in the ON DRT condition than in the OFF DRT one, we performed additional intergroup analyses between the early PD group in the OFF condition and HC. Regardless of the type of performance we analyzed (categorical or continuous ratings), we failed to find any significant differences (*p*>.2 for all the comparisons).

#### 3.4 Version A vs. B comparisons

No significant difference was found between the percentages of correct responses for Versions A and B, χ^2^(7) = 8.00, *p* = .2, in the HC group.

#### 3.5 Correlations between neuropsychological, psychiatric, and emotional prosody recognition data

No significant correlation was observed in any of the groups between vocal emotion recognition performances and the scores on the neuropsychological tests, age, level of education, or disease duration (*p*>.05 for all comparisons). Similarly, no significant correlation was observed in any of the groups between vocal emotion recognition performances and the scores on the psychiatric tests, notably the MADRS and the STAI (*p*>.05 for all comparisons).

## Discussion

The aim of the present study was to clarify the possible role of the BG and the nigrostriatal and mesolimbic dopaminergic pathways in emotional prosody recognition. We compared the performances of PD patients in the early and advanced stages of the disease with those of HC using an original emotional prosody (onomatopoeias) recognition paradigm. In addition, we compared the performances of the early PD patients ON and OFF DRT on the same task. Although the basic emotional prosody recognition procedure had previously been used in studies with patients (depressed patients and PD patients undergoing STN deep brain stimulation (DBS) [6], [27]), this was the first time that stimuli drawn from the MAV [36] (i.e., onomatopoeias) had been used with PD patients. This novel methodology proved to be highly sensitive to emotional bias in the PD population, and yielded patterns of results that mainly confirmed our operational hypotheses.

The *first statistical model* we built included the early PD ON, advanced PD and HC groups, in order to test our prediction that, whichever stage of the disease they were at (early or advanced), the PD patients on DRT would perform more poorly than HC on the emotional prosody task. We did indeed find that both the advanced PD and early PD patients in the ON condition exhibited impaired emotional prosody recognition, especially for the negative emotions (i.e., *fear*, *anger*, *disgust*), as well as an emotional bias reflected in higher ratings on nontarget scales: both the advanced PD and early PD ON patients gave significantly higher ratings on the *Anger* scale when they listened to *fear* stimuli (Fig. 1 Panel B & Table 4). The *second model* included the early PD patients in the ON versus OFF conditions, as we postulated that the toxicity of dopatherapy for emotional functions in the early stages of the disease would mean that the early PD patients performed more poorly on the recognition of emotional bursts in the ON condition than in the OFF one. The results for categorical ratings (i.e., percentages of correct responses; first level of analysis) revealed a significant difference between the early PD patients in the ON versus OFF conditions for the overall recognition score, as well as for the *anger*, *disgust*, and *fear* recognition subscores (Table 3). The investigation of continuous ratings (second level of analysis) revealed that the early PD patients provided significantly higher ratings on the *Anger* scale when they listened to *fear* in the ON condition than in the OFF one (Fig. 1 Panel D & Table 4). These intragroup results were reinforced by intergroup results revealing specific emotional impairment in the early PD patients in the ON DRT condition (but not the OFF one), compared with both the advanced PD patients and the HC (for the ratings on the *Anger* scale when they listened to *fear*, Fig. 1 Panel B, on the *Fear* scale when they listened to *anger*, Fig. 1 Panel A, and on the *Disgust* scale when they listened to *fear*, Fig. 1 Panel C).

### Control tasks

The patient and HC groups were randomly selected and matched for age and education level in order to avoid specific biases. All the participants were deemed to have normal hearing. The PD patients were divided into two distinct and homogeneous groups on the basis of disease duration and severity, age at the time of the interviews and dopa sensitivity. We also controlled for the homogeneity of our patient groups in terms of mood disorders. A handful of studies have suggested that patients with depression have impaired emotional prosody recognition (e.g., [27]). In the present study, both the early and advanced PD patients scored significantly higher on the depression scale (MADRS) and the anxiety scale (STAI-B) than the HC did (see Table 1). The presence of mood disorders is a classic observation in PD (for a review, see [38]). Nevertheless, we failed to find any significant correlation between these variables and the PD patients' emotional prosody recognition performances. As far as the neuropsychological variables are concerned, there was no significant difference between the early PD ON patients, advanced PD patients and HC on any variable (see Table 1). This failure to find any significant difference between the groups (especially between the advanced PD patients and the HC) on the neuropsychological tasks would, at first sight, appear to be relatively uncommon and warrant discussion. One major factor that could explain these results is that our advanced PD group was mainly composed of PD patients regarded as suitable candidates for STN DBS, and whose cognitive functions were therefore relatively intact. Clinicians classically observe that candidates for STN DBS are free of cognitive dysfunction in the pre-operative condition [6], [24], [25]. Indeed, it is one of the inclusion criteria for this type of surgery [39]. In addition, although we performed a conventional and quite exhaustive neuropsychological assessment, we can hypothesize that the tests we used were not sensitive enough to detect slight cognitive impairment in, say, decision making or reward learning. That said, as with mood, we failed to find any significant correlation between these neuropsychological variables and emotional prosody recognition performances.

### Limitations

There are several limitations that need to be acknowledged and addressed regarding the present study before we draw any inferences from our results. One major drawback of the study is that the advanced PD group was not studied in the OFF DRT condition. It would have been useful to look for differential performances between the early and advanced PD patients in both ON and OFF conditions. Indeed, as explained in detail further on in this Discussion, it limits the inferences that can be drawn about the potential dopamine overdose effect in this group of advanced PD patients. More specifically, we are unable to determine whether the emotional effects we observed were due to a potential dopamine overdose effect, lesions of the mesocorticolimbic pathway due to the progression of the disease, or a combination of the two. Nevertheless, we chose not to investigate the advanced PD patients in an OFF condition for obvious ethical considerations. In addition, we are inclined to think that advanced PD patients in an OFF condition would be unable to perform the cognitive and emotional tasks (lasting about 90 min) in the best conditions, thus making it well-nigh impossible to compare their performances with those of early PD patients in the same OFF condition. The second limitation concerns the neuropsychological and mood assessments undergone by the early PD group in the OFF condition. As the only assessment we administered to the early PD patients in the OFF condition was the MDRS, we were not able to test the dopamine overdose effect in the cognitive domain, or to find out whether the depressed symptoms were more (or less) intense in the OFF condition than in the ON one. We were therefore unable to investigate the influence of depressed mood on the emotional prosody performances of the early PD group in the OFF condition. This influence will need to be properly tested in future studies. That said, as we failed to find a significant correlation between depressed mood and emotional prosody processing in either the advanced PD and HC groups or the early PD ON group, we can speculate that early PD patients would display a similar pattern in the OFF condition. Third, it is important to note that all the PD patients (advanced and early ON) were not only on levodopa, but also on noncontrolled release dopamine agonists (ropinirole and/or pramipexole). Even though the OFF dopa assessments were performed 18–20 hours after the last medication intake, and even though we were thus beyond the duration of action of both the levodopa and the dopamine agonists, the latter have a longer plasma half-life than levodopa, which may have influenced the data. Careful account will have to be taken of this variable account in future studies exploring the emotional effects of anti-parkinsonian medication.

### BG and emotional prosody processing

The results showing that both the advanced PD patients and the early PD patients in the ON condition exhibited impaired emotional prosody recognition, especially for negative emotions (i.e., *fear*, *anger*, *disgust*), replicated previous findings reported in the literature, confirming that the BG are involved in emotional prosody processing. To date, studies have consistently observed impaired vocal emotion recognition in PD patients, compared with matched HC (for a review, see [8], [9]). BG involvement in emotional processing has also been documented in patient, lesion and fMRI studies (e.g., [2], [3], [40]). Paulmann and collaborators [40] suggested that the BG are involved in integrating emotional information from various sources. For instance, they are thought to play a functional role in comparing acoustic speech characteristics, such as perceived pitch, duration and intensity (i.e., prosodic information), and emotional semantic information. Patients with BG impairments therefore suffer from emotional speech deficits because they cannot perform the integration needed to decode emotional prosody [40]. Kotz and Schwartze [4] also recently underlined the functional role of the BG in decoding emotional prosody by suggesting that these deep structures are involved in the rhythmic aspects of speech decoding. More recently still, Péron and colleagues [7] modeled the functional specialization and integration of the BG in emotion processing, postulating that the BG coordinate neural patterns, either synchronizing or desynchronizing the activity of the different neuronal populations responsible for specific emotion components. In the context of vocal emotion recognition, for example, the BG would act as a marker for the transiently connected neural network (i.e., amygdala, auditory cortices, and orbitofrontal cortex) that subserves emotional prosody processing. If the co-activation across different neuronal populations is recurrent or functionally important, the BG-mediated synchronization presumably increases the weight of the synaptic connections within the network. According to this model, the BG play the role of neural rhythm organizer at the cortical and subcortical levels in emotional processing, thus explaining why they are sensitive to both the temporal and the structural organization of events [7].

### Dopaminergic pathways, deleterious effect of dopatherapy in the early stages of PD, and emotional prosody processing

The second pattern of results (i.e., emotional impairment only observed in the early PD ON group) enables us to draw two major inferences. First, dopaminergic pathways appear to be involved in emotional prosody processing. Second, dopatherapy appears to have a deleterious effect in the early stages of the disease.

Regarding the involvement of the dopaminergic pathways in emotional prosody processing, there is now a substantial body of evidence to support this hypothesis (for a review, see [41]). It should, however, be noted that, to the best of our knowledge, all the studies that have so far explored the involvement of the dopaminergic system in emotion processing in humans have used the facial, rather than the auditory, modality. This involvement can be tested in humans by manipulating dopamine agonists and antagonists. A study by Lawrence and colleagues [42] demonstrated that the recognition of angry facial expressions was diminished following the administration of a dopamine antagonist that blocked dopamine receptors. Similarly, an fMRI study of healthy individuals who had been given a dopaminergic antagonist revealed reduced activity of several limbic regions (amygdala, hippocampus, anterior cingulate cortex) during the perception of unpleasant images [43]. Another fMRI study reported increased amygdala activity during the perception of facial expressions of fear and anger after participants had received an agonist that increased the release of dopamine and inhibited its reuptake [44]. More recently, yet another fMRI study showed reduced bilateral amygdala activation during an emotional facial expression matching task in participants who had been administered levodopa [45]. Data suggesting dopamine involvement in emotional processes have also come from clinical studies of patients with neurological pathologies resulting in disturbed dopaminergic systems. Emotional prosody disorders have been reported in schizophrenia, autism, attention-deficit hyperactivity disorder, and Huntington's disease (for a review, see [9]). Finally, the fact that we only observed effects for negative emotions in the conditions where dopamine was manipulated is initially rather surprising, given that dopamine activations have been shown to be driven primarily by reward (i.e., positively valenced or pleasant stimuli [46]). That being said, changes in mesolimbic dopamine neurotransmission have been found to modify behavioral responses to a variety of environmental stimuli associated with reward behaviors (for a review, see [47]), not least reward prediction error signals [46]. In this context, prediction error signals, or *risks* could very well be linked to the recognition of negative emotions, and the dichotomy between reward/approach (presumably linked only to positive emotions) and punishment/avoidance (presumably linked only to negative emotions) is perhaps far more complex than it might first appear.

The intragroup results showed that the early PD patients performed significantly worse on *anger*, *disgust* and *fear* recognition in the ON condition than in the OFF one. In addition, they provided higher ratings on nontarget scales (e.g., the *Anger* scale when listening to *fear* stimuli) in the ON condition than in the OFF one. Together with the intergroup results showing that emotional disturbances were restricted to the early PD ON group, these results may have reflected an affective disturbance stemming from medication in the early stages of PD. As we explain in the Introduction, and even if it is important to acknowledge that neuroimaging data providing objective evidence of different patterns of dopaminergic degeneration in the early and advanced groups are missing from the present study, it is now well documented that VTA-innervated structures (mesocorticolimbic pathway) are less affected in the early stages of PD than the nigrostiatal pathway [48]. As the pathology progresses, VTA degeneration and the dopamine deficiency of its efferent structures increase. Our results seem to point to hyperstimulation by DRT of the mesocorticolimbic pathway, thus explaining the emotional disturbance observed in the early PD ON group. We can surmise that the advanced PD group performed slightly better than the early PD ON group because the DRT partially replenished their affected mesocorticolimbic pathway, instead of overdosing it, as it would appear to do in the earlier stages of the disease. Our results therefore seem to suggest the existence of the dopamine overdose effect [15], whereby the administration of dopaminergic medication to PD patients replenishes their dopamine-depleted circuits, but seemingly overdoses those that are still relatively intact. This effect has already been demonstrated for cognitive functions using a reward learning paradigm [10], but the present study is the first to suggest this effect in emotional prosody processing. Finally, these results also appear to confirm that the mesocorticolimbic pathway (VTA-innervated regions) is more closely involved in emotional prosody processing than the nigrostiatal pathway (e.g., dorsal striatum).

In conclusion, our results seem to confirm the hypothesis that the BG are involved in emotional prosody processing, and suggest that the dopaminergic system is involved, too. They have, however, to be confirmed with a larger sample of PD patients. In addition, further evidence needs to be gathered from PD patients, in particular using functional neuroimaging, to establish a correlation between emotional prosody processing and the mesocorticolimbic dopaminergic system, possibly by using specific metabolic markers. These results also underline the need for clinicians to look out for the emotional effects of dopaminergic treatment, at least during the early stages of the disease.

## Supporting Information

Appendix S1 **Computer interface for the original emotional prosody (onomatopoeias) recognition paradigm.**
(DOCX)
