# Supplementary material for: Effect of Dopamine Therapy on Nonverbal Affect Burst Recognition in Parkinson's Disease
Source: PLoS One. 2014 Mar 20;9(3):e90092. doi: 10.1371/journal.pone.0090092 (PMC3961247; doi:10.1371/journal.pone.0090092)

# **Appendix S1: Computer interface for the original emotional prosody (onomatopoeias) recognition paradigm**


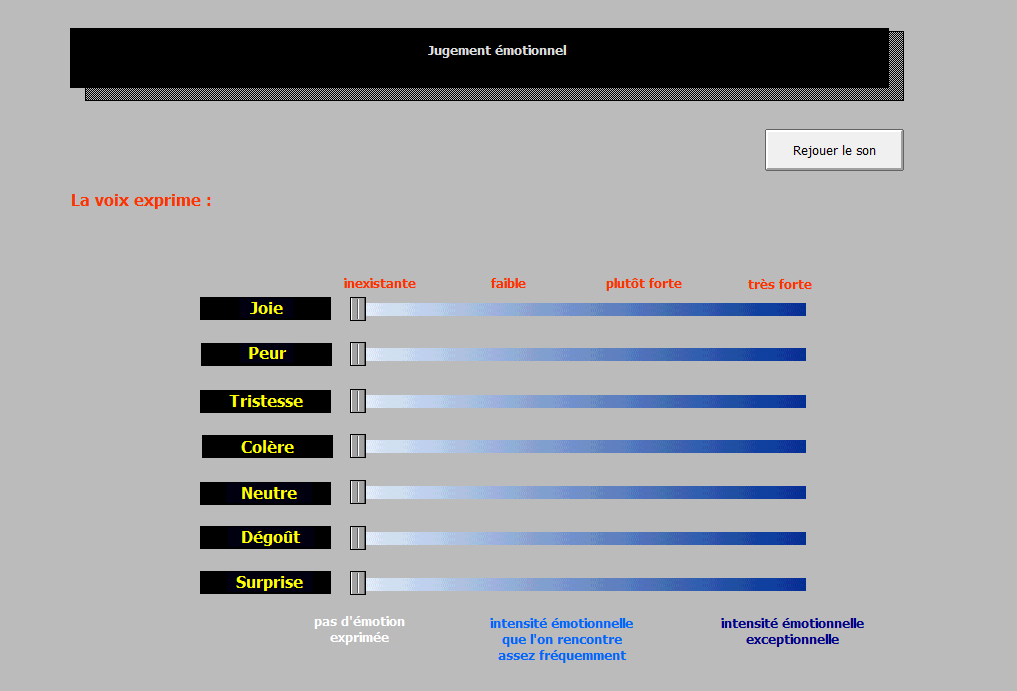

Supplement: Appendix S1 — Computer interface for the original emotional prosody (onomatopoeias) recognition paradigm. (DOCX) [file pone.0090092.s001.docx]
